# The Mediating Effect of Trunk Impairment on Quality of Life in Subacute Stroke Survivors

**DOI:** 10.1155/nrp/9547963

**Published:** 2026-04-02

**Authors:** Su-Ju Tsai, Chun-Chang Lin, Chieh-Tsung Yen, Chia-Chi Li, Hsiang-Chu Pai

**Affiliations:** ^1^ Department of Physical Medicine and Rehabilitation, Chung Shan Medical University, Taichung City, Taiwan, csmu.edu.tw; ^2^ Department of Physical Medicine and Rehabilitation, Chung Shan Medical University Hospital, Taichung City, Taiwan, csh.org.tw; ^3^ Department of Nursing, Chi Mei Hospital, Chiali, Tainan City, Taiwan; ^4^ Department of Neurology, Dalin Tzu Chi Hospital, Buddhist Tzu Chi Medical Foundation, Chiayi County, Taiwan, tzuchi.com.tw; ^5^ Department of Nursing, Chung-Shan Medical University Hospital, Taichung City, Taiwan, csmu.edu.tw; ^6^ Department of Nursing, Chung Shan Medical University, Taichung City, Taiwan, csmu.edu.tw

**Keywords:** quality of life, stroke survivors, trunk impairment, trunk performance

## Abstract

**Objective:**

This study aimed to clarify the determinants (e.g., age, sex, education level, stroke type, and disease severity) of trunk impairment and quality of life (QoL) among stroke survivors and to examine whether trunk performance mediates the relationship between the National Institutes of Health Stroke Scale (NIHSS) and QoL.

**Method:**

A quantitative, correlational design was employed and conducted between August 2023 and March 2025. Participants were stroke survivors recruited from the rehabilitation wards of three hospitals in Taiwan. The questionnaire included four measures: demographic data (including stroke type), the NIHSS (disease severity), the Trunk Impairment Scale (trunk performance), and the Stroke Impact Scale (QoL). Structural equation modeling with partial least squares (PLS‐SEM) was used to examine the relationships among the variables. Model fit was assessed using the normed fit index (> 0.90) and the standardized root‐mean‐square residual (< 0.08).

**Results:**

A total of 160 stroke survivors participated, with a mean age of 63.04 years (SD = 13.29). Age (*β* = −0.145, *p* = 0.020) and disease severity (*β* = −0.60, *p* < 0.001) were significantly and negatively associated with trunk performance. Disease severity was also significantly and negatively associated with QoL (*β* = −0.196, *p* < 0.001). Trunk performance partially mediated the relationship between disease severity and QoL (*β* = 0.647, *p* < 0.001). Ischemic stroke type was significantly and positively associated with QoL (*β* = 0.194, *p* = 0.049). Collectively, disease severity, trunk performance, and ischemic stroke type explained 64.0% of the variance in QoL (*Q* ^2^ = 0.358, *f* ^2^ = 0.807), indicating strong predictive power.

**Conclusion:**

Stroke survivors with greater disease severity, poorer trunk performance, and hemorrhagic stroke experience lower QoL. Older individuals are more likely to exhibit trunk impairment. Notably, greater disease severity may lead to poorer trunk performance, which in turn contributes to reduced QoL. Rehabilitation interventions should focus on strengthening trunk control and stability in stroke survivors.

**Summary**
• What is already known?
◦ Neurological deficits in stroke survivors significantly affect QoL, and the NIHSS serves as a predictive tool for QoL in these patients.
◦ Empirical evidence demonstrates that better trunk function is associated with improved ADL performance.
◦ Previous studies’ findings on the relationships among the demographic characteristics of stroke survivors, disease severity, trunk impairment, and quality of life have been inconsistent.
• What does this study add?
◦ Older stroke survivors are more likely to experience trunk impairment.
◦ Survivors with hemorrhagic stroke had a poorer quality of life than those with ischemic stroke.
◦ Disease severity, trunk performance, and stroke type were significant predictors of overall quality of life.
◦ Trunk performance partially mediated the relationship between disease severity and quality of life.
• Patient or public contribution
◦ No public or patient contribution outside of participation for data collection purposes.
• Reporting method
◦ The authors of this manuscript have adhered to relevant EQUATOR guidelines, which are based on the STROBE cross‐sectional reporting method.

## 1. Introduction

Studies have shown that 96.4% of stroke survivors experience trunk impairment [1], and the role of nurses in rehabilitation wards has gained increasing attention [2]. From a nursing care perspective, the demographic characteristics of stroke survivors, such as age, sex, and stroke type, should be considered to address individual care needs. However, findings on the relationships among the demographic characteristics of stroke survivors, disease severity, trunk impairment, and quality of life (QoL) have been inconsistent. These inconsistencies highlight the need for further clarification regarding the impact of individual demographic characteristics and disease severity on trunk function in stroke survivors.

Neurological deficits in stroke survivors significantly affect QoL, and the NIHSS serves as a predictive tool for QoL in these patients [3]. Braga et al. emphasized that stroke severity is a critical factor in identifying individuals at risk of health‐related QoL decline [4]. Collectively, these studies underscore the significant impact of disease severity on the QoL of stroke survivors. Additionally, stroke survivors often experience varying degrees of trunk impairment. Kong and Krishnan suggested that more severe strokes result in greater trunk impairment [1]. Similarly, Martins et al. highlighted that higher National Institutes of Health Stroke Scale (NIHSS) scores are associated with poor trunk control [5], which may decrease the ability to perform daily activities [6, 7]. Moreover, empirical evidence demonstrates that better trunk function is associated with improved activities of daily living (ADL) performance [6, 7]. Trunk impairment, by contrast, reduces ADL independence [7] and QoL [8]. Such impairments affect balance, sitting posture, and walking ability, further diminishing QoL. These findings indicate that disease severity affects trunk performance in patients with stroke, which in turn affects QoL. Therefore, whether the impact of disease severity on ADL or QoL is fully or partially mediated by trunk function requires further investigation.

Notably, Aaslund et al. identified age as a predictor of walking ability among patients with stroke while excluding the influence of NIHSS scores [9]. Other studies have reported conflicting findings. For example, Alawieh, Zhao, and Feng suggested that advanced age and female sex are major socioeconomic factors affecting stroke recovery [10], whereas Pellicciari et al. found that neither age nor sex significantly predicts ADL among patients with stroke [7]. Because walking ability is affected by trunk function [11], age may not directly affect ADL in patients with stroke; rather, it may first affect trunk performance and then indirectly influence ADL or QoL. In addition to disease severity, studies have also investigated the effects of stroke type on QoL, although findings remain inconsistent. For example, Abd Ali [12] and Unibaso‐Markaida et al. [13] reported that patients with hemorrhagic stroke exhibit poorer QoL across all domains compared to those with ischemic disease. Contrastingly, Sridhar et al. found that patients with hemorrhagic stroke had better QoL than those with ischemic stroke [14].

Overall, the literature indicates that disease severity adversely affects trunk performance, leading to impairments in balance, gait, and functional ability in patients with stroke, which may result in decreased QoL. However, the relationships among patient demographic characteristics, disease severity, trunk performance, and QoL remain unclear. Structural equation modeling with partial least squares (PLS‐SEM) performs separate regressions of each dependent construct in the structural model on its associated independent constructs, using an iterative process that accounts for the entire model structure. As a result, PLS‐SEM is suitable for the analysis of complex models and small sample sizes. In addition, PLS‐SEM is considered a preferred approach for estimating mediation and conditional process models [15]. Therefore, this study employs PLS‐SEM to clarify these relationships and further investigate the mediating effect of trunk performance on QoL in patients with stroke.

### 1.1. Hypotheses

This study builds upon previous findings, considering NIHSS scores and demographic variables as independent variables, trunk performance as a mediator, and QoL as the outcome. The research hypotheses are as follows:

 H1: NIHSS negatively and significantly affects trunk performance.
 H2: NIHSS negatively and significantly affects QoL.
 H3: Trunk performance positively and significantly affects QoL and mediates the relationship between NIHSS score and QoL.
 H4: Demographic variables (e.g., age, sex, and education level) and stroke type affect trunk performance and QoL.

## 2. Methods

### 2.1. Study Design

A cross‐sectional study design with mediation analysis was used.

### 2.2. Study Setting and Sampling

Stroke survivors were recruited from rehabilitation wards in three hospitals in Taiwan. Inclusion criteria were (a) hospitalization in a rehabilitation ward; (b) age > 20 years; and (c) willingness to participate. Patients with acute stroke or unstable medical conditions were excluded, including patients with current urinary tract infections, pneumonia, diabetes, kidney disease, or other conditions that could affect their current activities or QoL. Based on a recommended sample‐size‐to‐parameters ratio (*q* = 20:1) [16], with *q = *7, a minimum sample size of 140 was required. This study included 160 stroke survivors, satisfying the sample size requirement.

### 2.3. Data Collection

The study’s questionnaire comprised four measures: demographic characteristics, NIHSS, Trunk Impairment Scale (TIS), and Stroke Impact Scale (SIS). Five experts (two nursing professors, two physical therapists, and one rehabilitation physician) reviewed the questionnaire content. Using a 4‐point scale ranging from 1 (*not relevant*, *not clear*, *not simple,* or *doubtful*) to 4 (*very relevant*, *very clear*, *very simple*, or *meaning is clear*), each expert rated the relevance, clarity, simplicity, and ambiguity of each item [17]. The content validity indices for all instruments exceeded 0.87, which is above the widely accepted cutoff value of 0.80 for adequate content validity [18].

#### 2.3.1. TIS

Trunk performance was assessed using the 17‐item TIS [19, 20], divided into three domains: static postural balance (7 points), dynamic balance (10 points), and coordination (6 points). Each item was scored on scales of 0–1, 0–2, or 0–3. Total scores ranged from 0 to 23, with higher scores indicating better trunk performance. Cronbach’s *α* for TIS in this study was 0.87.

#### 2.3.2. NIHSS

The NIHSS (15 items) was used to assess neurological impairment in stroke survivors. It evaluates consciousness, extraocular movement, visual field loss, facial palsy, motor strength, sensory loss, neglect, language, dysarthria, and ataxia [19]. Scores range from 0 to 42, with higher scores indicating greater severity. Stroke severity categories were *mild* (1–4), *moderate* (5–14), and *serious* (≥ 15) [19].

#### 2.3.3. SIS

The SIS‐16 (Version 3.0) was used to assess QoL [20, 21], and the Chinese version was used with permission from the original author. It includes 16 items across three domains: hand function (1 item), mobility (8 items), and ADL (7 items). Items were scored from 1 (*could not do at all*) to 5 points (*not difficult at all*). Total scores were standardized to a range of 0–100, with lower scores indicating a high impact on QoL [20, 21]. In this study, the scale had a Cronbach’s *α* of 0.97.

### 2.4. Ethical Considerations

The Institutional Review Board (IRB) of Chung Shan Medical University Hospital (Approval no. CS1‐2307; date: May 2, 2023) approved the study design and procedures. Written informed consent was obtained after participants were briefed on the study purpose. All participants filled out the questionnaire anonymously. This study was conducted from August 2023 to March 2025.

### 2.5. Data Analysis

A two‐phase data analysis was conducted. First, IBM SPSS Statistics Version 26 (IBM, Armonk, NY, USA) was used to perform an independent sample *t*‐test and Pearson’s correlation test to examine relationships between the demographic variables (e.g., age, sex, education, and stroke type), NIHSS, TIS, and the outcome variable (SIS). Second, complex model linkages between variables can be clarified using a PLS‐SEM analysis [22]. Therefore, we conducted SEM with PLS and bootstrapping to test the hypothetical model using SmartPLS software (v.4.1.0.6) [23]. Categorical variables were dummy‐coded (0–1) before inclusion in the model [24].

Three indicators—composite reliability (CR > 0.70), indicator reliability (loading > 0.70), and average variance extracted (AVE > 0.5)—were used to assess the measurement model’s validity and reliability [25]. The Fornell–Larcker criterion was used to assess the discriminant validity of each construct [25, 26]. The coefficients of determination (*R* ^2^), predictive relevance (Q^2^), effect size (*f* ^2^), and significance of path coefficients (*p* < 0.05) were used to evaluate the structural model’s validity. According to Hair et al., *R* ^2^ values of endogenous latent constructs can be interpreted as 0.25 (weak), 0.50 (moderate), and 0.75 (substantial). *Q* ^2^ values indicating predictive relevance of endogenous constructs greater than 0, 0.25, and 0.50 represent small, medium, and large predictive accuracy of the PLS path model, respectively [24]. It is also recommended that *f* ^2^ values of 0.02–0.14, 0.15–0.34, and 0.35 correspond to small, medium, and large effect sizes, respectively [24]. To assess the model fit, the normed fit index (NFI > 0.90) and standardized root‐mean‐square residual (SRMR < 0.08) were employed [16, 27].

## 3. Results

### 3.1. Participants’ Demographics and Variables Analysis

Initially, 164 stroke survivors were invited to participate in the study; four did not complete the questionnaire. The final analysis included 160 patients, yielding a response rate of 97.56% with no missing data. As shown in Tables 1 and 2, the mean age was 63.04 years (SD = 13.29), with 103 participants (64.4%) being male. Of the participants, 73.8% had a high school education or less, and the majority had ischemic stroke (70.0%). Age and NIHSS showed significant negative correlations with TIS (*r* = −0.20, *p* = 0.010; *r* = −0.61, *p* < 0.01), respectively, and NIHSS was significantly negatively correlated with SIS (*r* = −0.62, *p* < 0.01). Results from the independent *t*‐test indicated that stroke type significantly correlated with SIS (*t* = −2.573, *p* = 0.011). However, no significant differences were observed by sex or education level in TIS or SIS. The original mean SIS was 53.66 (SD = 19.70), and after standardization to a 0–100 scale, the mean increased to 58.34 (SD = 30.78), indicating a moderate QoL among patients. The mean TIS score was 12.10 (SD = 6.24), and based on the recommended cutoff value of 16.5 (with a moderately accurate AUC of 0.90 [0.83–0.98]) [28], 118 participants (73.8%) scored ≤ 16.5, whereas 45 (26.3%) scored > 16.5, indicating that most stroke survivors in the sample experienced trunk impairment. The average NIHSS score was 4.91 (SD = 4.93), indicating moderate disease severity.

**TABLE 1:** Comparison of the performance of different demographic and disease variables in TIS and SIS (*n* = 160).

Variables	*n*	%	TIS			SIS		
			M ± SD	t or r	*p*	M ± SD	t or r	*p*
Age (year)	160	100	—	−0.20^∗^	0.010	—	−0.141	0.076
Sex	—	—	—	0.81	0.419	—	0.17	0.869
Male	103	64.4	12.40 ± 6.24	—	—	59.15 ± 31.10	—	—
Female	57	35.6	11.56 ± 6.27	—	—	58.31 ± 30.46	—	—
Education	—	—	—	−1.44	0.152	—	−1.14	0.255
≦ High school	118	73.8	11.68 ± 6.37	—	—	56.19 ± 31.23	—	—
≧ College or university	42	26.3	13.29 ± 5.77	—	—	63.50 ± 29.34	—	—
Stroke type	—	—	—	−1.30	0.197	—	−2.57^∗^	0.011
Hemorrhages	48	30.0	11.13 ± 6.19	—	—	49.45 ± 27.70	—	—
Ischemic	112	70.0	12.52 ± 6.25	—	—	62.88 ± 31.27	—	—
NIHSS	160	100	—	−0.61^∗∗^	< 0.01	—	−0.62^∗∗^	< 0.01

Abbreviations: NIHSS = National Institutes of Health Stroke Scale, SIS = Stroke Impact of Scale, TIS = Trunk Impairment of Scale.

^∗^ *p* < 0.05.

^∗∗^ *p* < 0.01.

**TABLE 2:** Descriptive statistics, reliability, and valid for the measurement model (*N* = 160).

Variables	Descriptive statistics			Reliability and validity		
	Range score	M	SD	Loading	CR	AVE
TIS	0–23	12.10	6.24	—	0.81	0.72
Static postural balance	0–7	4.93	2.25	0.88^∗∗∗^	—	—
Dynamic balance	0–14	4.49	3.18	0.86^∗∗∗^	—	—
Coordination	0–6	2.68	1.87	0.80^∗∗∗^	—	—
SIS	0–100	58.84	30.78	—	0.94	0.90
Hand function	1–5	2.76	1.57	0.93^∗∗∗^	—	—
Mobility	8–40	22.94	9.41	0.97^∗∗∗^	—	—
Activities of daily living	7–35	27.95	9.43	0.95^∗∗∗^	—	—
NIHSS	0–24	4.91	4.93	Single item	1.00	1.00
Age (year)	—	63.04	13.29	Single item	1.00	1.00
Stroke type (ischemic)	—	—	—	Single item	1.00	1.00

Abbreviations: AVE = average variance extracted, CR = composite reliability, NIHSS = National Institutes of Health Stroke Scale, SIS = Stroke Impact of Scale, TIS = Trunk Impairment of Scale.

^∗∗∗^ *p* < 0.001.

### 3.2. Reliability and Validity of the Measurement Model

NIHSS, age, and stroke type were included in the model due to their significant correlations with TIS or SIS. As shown in Table 2, these three variables (NIHSS, age, and stroke type) yielded CR and AVE values of 1.00, which resulted from treating the total score or category variable as a single indicator. Additionally, the latent variables TIS and SIS achieved CR values of 0.81 and 0.94, along with AVE values of 0.72 and 0.90, respectively. These values meet the recommended thresholds of CR > 0.70 and AVE > 0.50 [25]. Furthermore, factor loadings for both latent variables exceeded 0.70, ranging from 0.80 to 0.97 (*p* < 0.001), indicating adequate convergent validity, internal consistency, and reliability for the measurement scales [25].

As presented in Table 3, the square root of AVE for all constructs was greater than the correlations with other constructs, indicating that discriminant validity was achieved [25, 26].

**TABLE 3:** The validity of the structural model (*N* = 160).

Variables	*R* ^2^	*Q* ^2^	*f* ^2^	Effects	Fronell–larcker criterion (correlation of latent constructs)				
					1	2	3	4	5
1. Age	—	—	—	—	**1.00**	—	—	—	—
2. Stroke type	—	—	—	—	0.27	**1.00**	—	—	—
3. NIHSS	—	—	—	—	0.09	−0.23	**1.00**	—	—
4. TIS	0.398	0.382	0.629	Medium to large	−0.20	0.11	−0.61	**0.85**	—
5. SIS	0.640	0.358	0.807	Medium to large	−0.16	0.20	−0.61	0.78	**0.95**

*Note*: The diagonal elements in bold in the correlation of constructs matrix are the square roots of the average variance extracted (AVE).

Abbreviations: NIHSS = National Institutes of Health Stroke Scale, SIS = Stroke Impact of Scale, TIS = Trunk Impairment of Scale.

### 3.3. Assessment of the Structural Model

According to Hair (2020), a dummy independent variable is defined as a variable with two distinct levels coded as 0 and 1. Dummy‐coded variables enable the use of independent variables not measured on an interval scale to predict a dependent variable (p. 413) [24]. Therefore, dummy‐coded indicators can be included in PLS‐SEM [29]. In this study, the independent *t*‐test indicated that stroke type was significantly associated with SIS; thus, stroke type was included in the model by coding hemorrhagic stroke as 0 and ischemic stroke as 1.

**FIGURE 1:**
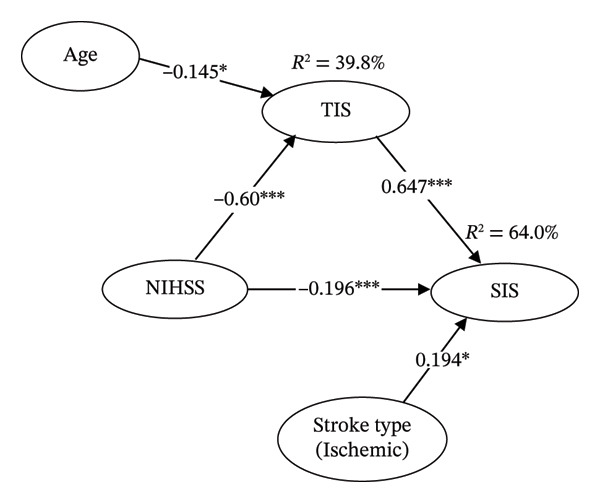
Results of the model of the relationships between research variables (SRMR = 0.044 and NFI = 0.91). Note: NIHSS = National Institutes of Health Stroke Scale; TIS = Trunk Impairment of Scale; SIS = Stroke Impact of Scale; ^∗^ *p* < 0.05, ^∗∗∗^ *p* < 0.001; SRMR = standardized root‐mean‐square residual; NFI = normed fit index.

Figure 1 presents the construct variables’ path coefficients and model analysis results. The model fit the data well, with an SRMR of 0.044, below the threshold of 0.080, and an NFI of 0.91, meeting the required threshold [16, 27]. The results indicate that NIHSS was a significant negative predictor of TIS (*β* = −0.60 [95% CI = −0.686 to −0.510], *p* < 0.001), and age was also a significant negative predictor of TIS (*β* = −0.145 [95% CI = −0.271 to −0.024], *p* = 0.020). Additionally, NIHSS showed a significant negative association with SIS (*β* = −0.196 [95% CI = −0.304 to −0.090], *p* < 0.001), and positively predicted SIS through TIS mediation (*β* = 0.647 [95% CI = 0.535 to 0.749], *p* < 0.001). Overall, age and NIHSS explained 39.8% of the variance in TIS (*Q* ^2^ = 0.382, *f* ^2^ = 0.629). Furthermore, stroke type (ischemic stroke) was a significant positive predictor of SIS (*β* = 0.194 [95% CI = 0.002 to 0.389], *p* = 0.049). Finally, TIS, NIHSS, and stroke type accounted for 64.0% (*Q* ^2^ = 0.358, *f* ^2^ = 0.807) of the total variance in SIS (Table 3). These findings fully support H1, H2, and H3 and partially support H4 (e.g., age and stroke type).

## 4. Discussion

This study aimed to identify potential factors predicting QoL among stroke survivors during the subacute phase, with disease severity (NIHSS) as the primary outcome measure. Furthermore, we examined whether trunk performance mediates the relationship between disease severity (NIHSS) and QoL. Certain demographic variables (e.g., age and stroke type) were also included in the predictive model.

Our results indicated that the NIHSS score and age were significantly negatively correlated with trunk performance, with greater disease severity and older age presenting with poor trunk performance. This result is consistent with previous studies indicating that the greater the severity of stroke, the greater the trunk impairment [1, 5]. Furthermore, our study found that older survivors had worse trunk performance. Therefore, we suggest that special attention should be paid to older adult patients with stroke. Older adults (the more severe the disease) may not have sufficient control over their center of posture when sitting, which may lead to deviation or an unstable sitting posture. Nurses need to prevent patients with stroke from falling, especially if there is insufficient support when getting up, as they are more likely to be injured.

Initially, we found that the NIHSS (disease severity) had a significant negative correlation with stroke impact (QoL); that is, the more severe the disease, the worse the QoL. Additionally, TIS significantly and positively affected SIS. This finding indicates that trunk performance partially mediates the relationship between disease severity and QoL. These findings are consistent with studies indicating that TIS is an early predictor of ADL among patients with stroke [30, 31]. Furthermore, our findings support the suggestion by Sato and Ogawa that trunk function is an important key indicator of recovery in patients with stroke [11]. It has also been shown that disease severity affects the trunk control ability of patients with stroke, thereby leading to a decrease in their ability to perform daily activities [6, 7] and reduced QoL [8]. Therefore, trunk performance in patients with stroke is an important factor associated with QoL. Future longitudinal or interventional studies are needed to determine whether changes in trunk function are associated with changes in QoL. We suggest that rehabilitation ward nurses develop and design nursing interventions suitable for bedside implementation to strengthen and improve trunk function in patients.

Our study also revealed that having an ischemic stroke is significantly positively associated with QoL, with patients with ischemic stroke reporting better QoL than those with hemorrhagic stroke. This finding is consistent with previous studies [12, 13], which pointed out that the hemorrhagic type affects various aspects of the QoL of patients with stroke and is associated with a worse QoL than the ischemic type. However, this is different from the conclusion of a study by Sridhar et al. [14], which showed that patients with hemorrhagic stroke had a better QoL than those with ischemic stroke. The discrepancy may stem from participant differences; for instance, 64.4% of participants in our study were male, whereas Sridhar et al. included 80.6% males. Although our study found no significant sex differences in QoL, Sridhar et al. reported higher QoL scores among males. Future research should include larger, sex‐stratified samples and employ SEM analysis to explore these differences. Additionally, no significant differences in TIS or SIS were found across sex or educational level, suggesting similar functioning across demographic groups. Therefore, interventions targeting TIS and SIS may adopt universal approaches, whereas future research should explore other individual or contextual factors influencing these constructs.

In summary, the full model revealed that 39.8% of the variance in TIS among patients with stroke was explained by age and NIHSS. A *Q* ^2^ value of 0.382 and an *f* ^2^ value of 0.629 indicate a substantial effect size and moderate‐to‐high predictive accuracy, respectively [24]. Furthermore, all research variables accounted for 64.0% of the overall variance in SIS, indicating a substantial effect size (*Q* ^2^ = 0.358, *f* ^2^ = 0.807) and moderate‐to‐high predictive accuracy [24]. Thus, the proposed research paradigm was validated.

### 4.1. Limitation

This study had some limitations that need to be considered before interpreting the findings. First, the use of self‐reported SIS for QoL measures may introduce measurement bias; however, it is a common challenge in social science research. Findings should therefore be interpreted with caution. Future studies could incorporate objective measures or data from multiple sources to improve validity. Second, the cross‐sectional design cannot establish causal relationships. Although we employed robust statistical methods to identify predictors of QoL among stroke survivors and emphasized the importance of strengthening trunk function, experiments and longitudinal studies are needed to further validate and enhance our findings. Additionally, although we compared trunk performance and QoL across sexes and stroke type, we did not conduct multigroup SEM analyses. Future research should aim to balance the ratio of males and females and stroke types and perform multigroup SEM analyses to explore potential sex‐based differences more comprehensively.

## 5. Conclusion

This study enhances the understanding of stroke survivors’ QoL and its determinants. Using PLS‐SEM analysis, we found that NIHSS (disease severity) and trunk performance are significant predictors of QoL. Furthermore, trunk performance partially mediates the relationship between disease severity and QoL. These findings underscore the critical role of trunk function in improving QoL among stroke survivors.

## Author Contributions

Hsiang‐Chu Pai and Su‐Ju Tsai were responsible for the study conception and design. Chun‐Chang Lin, Chieh‐Tsung Yen, and Chia‐Chi Li performed the data collection. Hsiang‐Chu Pai was involved in data analysis, manuscript drafting, and critical revisions for important intellectual content.

## Funding

This study was funded by the National Science and Technology Council, Taiwan (NSTC112‐2314‐B‐040‐004).

## Ethics Statement

The Institutional Review Board (IRB) of Chung Shan Medical University Hospital granted the study’s permission and design (CSMUH No: CS1‐23077; date: May 2, 2023).

## Conflicts of Interest

The authors declare no conflicts of interest.

## Data Availability

The raw data that support the findings of this study are available upon reasonable request from the corresponding author.
